# Fatal Pulmonary Mucormycosis in a Renal Transplant Recipient: A Missed Opportunistic Infection

**DOI:** 10.7759/cureus.106886

**Published:** 2026-04-12

**Authors:** Tatiana Correa, Gregory Braden, Barbara Greco, Kenneth Brand, Yasin Obeidat, Umair Khan

**Affiliations:** 1 Department of Internal Medicine, University of Massachusetts Chan Medical School - Baystate, Springfield, USA; 2 Department of Nephrology, University of Massachusetts Chan Medical School - Baystate, Springfield, USA

**Keywords:** mucormycosis, opportunistic infection, pneumonia, renal transplant, transplant

## Abstract

Pulmonary mucormycosis has a high mortality rate if not diagnosed promptly. Early diagnosis and aggressive antifungal treatment, along with surgical debridement, can improve outcomes. We present a case of a 55-year-old African American man with end-stage renal disease who developed fatal pulmonary mucor seven months following his second deceased donor kidney transplant. This case highlights the pitfalls of empiric approaches to infections in immunocompromised hosts, assumptions based on the presence of concomitant organisms that may be present and the importance of accurate interpretation of serologic testing. The aim of this case report is to emphasize the critical importance of considering opportunistic infection in the differential diagnosis, as well as the need for early tissue diagnosis when solid organ transplant recipients present with pulmonary infections.

## Introduction

Kidney transplant recipients require long-term immunosuppressive therapy to prevent allograft rejection, a process in which the recipient’s immune system recognizes the transplanted kidney as foreign and mounts an immune response against it. While immunosuppression preserves graft function, it also weakens host defenses, predisposing patients to opportunistic infections or infections caused by organisms that would not have caused disease in immunocompetent patients. Among invasive fungal infections in kidney transplant recipients, *Candida* and* Aspergillus* species are the most common pathogens, with mucormycosis accounting for only 2.0% of cases [1].

*Mucor* species are widely present in the environment, primarily in soil, reproducing rapidly and releasing airborne spores that are ubiquitous contaminants in microbiology labs. In 30% of cases, pulmonary infections with mucor can be superimposed on bacterial pneumonia, leading to delayed diagnosis [2]. The definitive diagnosis of pulmonary mucormycosis (PM) requires tissue histology or culture. The mortality rate of mucor infections is very high, ranging between 30% and 100%, depending on the clinical presentation [3]. It tends to progress rapidly, making early diagnosis and initiation of effective therapy critical for a favorable outcome. We present a fatal case of PM, which evaded early diagnosis and treatment.

## Case presentation

A 55-year-old African American male with a history of end-stage renal disease secondary to hypertensive nephrosclerosis and a prior failed deceased donor kidney transplant in 2012 underwent a second deceased donor kidney transplant in February 2023 after 10 years of maintenance hemodialysis. His medical history included Roux-en-Y gastric bypass for obesity, tertiary hyperparathyroidism, diverticulosis, recurrent anal fissures, hyperlipidemia, and depression. Induction immunosuppression consisted of alemtuzumab and solumedrol, with maintenance therapy including tacrolimus and mycophenolate mofetil.

The post-transplant course was complicated by delayed graft function requiring hemodialysis for approximately three weeks. At one month post-transplant, the patient developed de novo donor-specific antibodies, underwent renal allograft biopsy, and was treated with seven sessions of apheresis and intravenous immunoglobulin (IVIG) for early antibody-mediated rejection. Prednisone was subsequently added to the immunosuppressive regimen. Follow-up allograft biopsy showed no rejection, and creatinine levels stabilized in the 2.8-3.3 mg/dL range. The patient had a brief admission three months post-transplant for acute kidney injury associated with diarrhea, which improved with volume repletion.

In August 2023, seven months post-transplant, the patient presented with ongoing diarrhea, fever, abdominal discomfort, and weakness. He reported one episode of hemoptysis but denied chest pain or shortness of breath. He had lost 25 pounds over the prior three months with reduced appetite and fatigue. On examination, he was febrile to 99.3°F with a blood pressure of 130/78 mmHg. Laboratory data are seen in Table 1.

**Table 1:** Laboratory data.

Lab Test	Patient Value	Reference Range
Hemoglobin	12.1 g/dL	13.7-17.1 g/dL
White Blood Cell Count	4.3 x10^3/µL	4.0-11.0 x 10^3/µL
Neutrophils	90.6%	44-76%
Bicarbonate	18 mmol/L	22-29 mmol/L
Anion Gap	17 mmol/L	4-7mmol/L
Serum Creatinine	6.7 mg/dL	0.7-1.2 mg/dL
Tacrolimus Level	12.0 ng/mL	5-20 ng/mL
Alkaline Phosphatase	130 U/L	40-129 U/L
Lipase	283 U/L	13-60 U/L
Beta-D-Glucan (Fungitell Assay)	31 pg/mL	<80 pg/mL
Galactomannan	0.02	0.0-0.49 index

**Figure 1:**
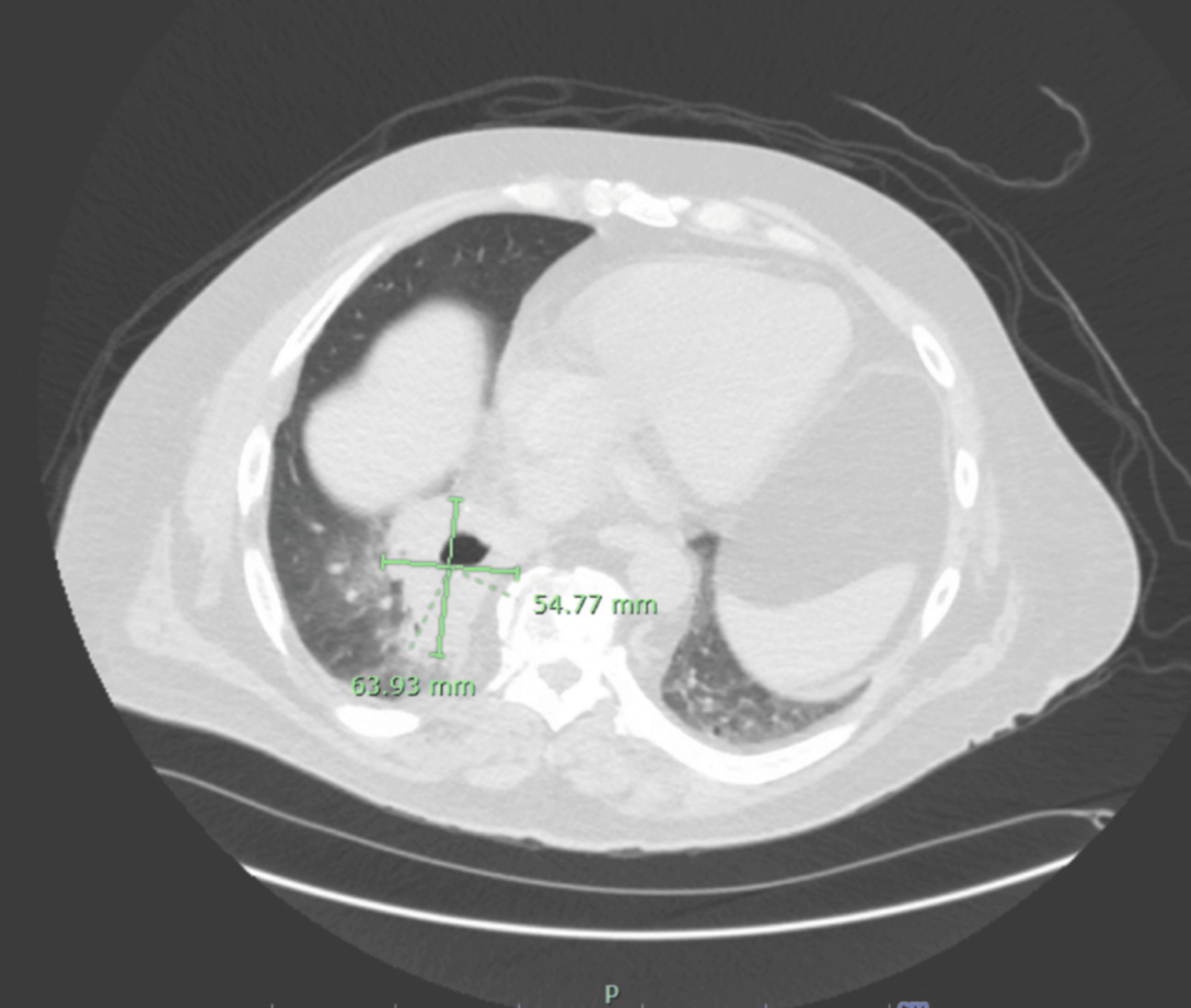
CT scan of the lung on day 1 showing a thick-walled cavitated lesion measuring 54.77 mm x 63.93 mm. Image obtained at our institution, Baystate Medical Center, and used with family consent.

A computed tomography (CT) scan of the abdomen and pelvis showed diverticulosis with mild thickening in the mid-ascending colonic wall. The CT scan also revealed a right lower lung (RLL) thick-walled 6.4 x 5.5 x 7.3 cm cavitary lesion containing an air-fluid level, suspicious for abscess (Figure 1). The patient was administered intravenous vancomycin and ampicillin/sulbactam and was admitted for further care. Mycophenolate was discontinued. Infectious disease was consulted on day 1.

An infectious workup, including a nasopharyngeal swab for polymerase chain reaction (PCR) identification of *Staphylococcus aureus* and *Mycobacterium* complex, as well as a viral panel including SARS-CoV-2, was unrevealing. Serologic antigen testing for *Histoplasma* and* Cryptococcus *was negative, and beta-D-glucan was 31 pg/mL (Fungitell assay), with a negative galactomannan of 0.02. Blood cultures were negative.

Bronchoscopy performed on hospital day 4 revealed *Streptococcus* species in bronchoalveolar lavage (BAL) washings from the right lower lobe. Additional BAL samples were obtained for acid-fast bacilli, fungal and anaerobic cultures, cytology, and *Legionella* testing; all were nondiagnostic. A 10-day course of piperacillin-tazobactam was administered for presumed *Streptococcus pneumoniae*. No tissue biopsy was obtained during bronchoscopy. Given the initial clinical improvement on piperacillin-tazobactam, the BAL growth of *Streptococcus*, and negative fungal cultures, the infectious disease service agreed with the continuation of therapy.

**Figure 2:**
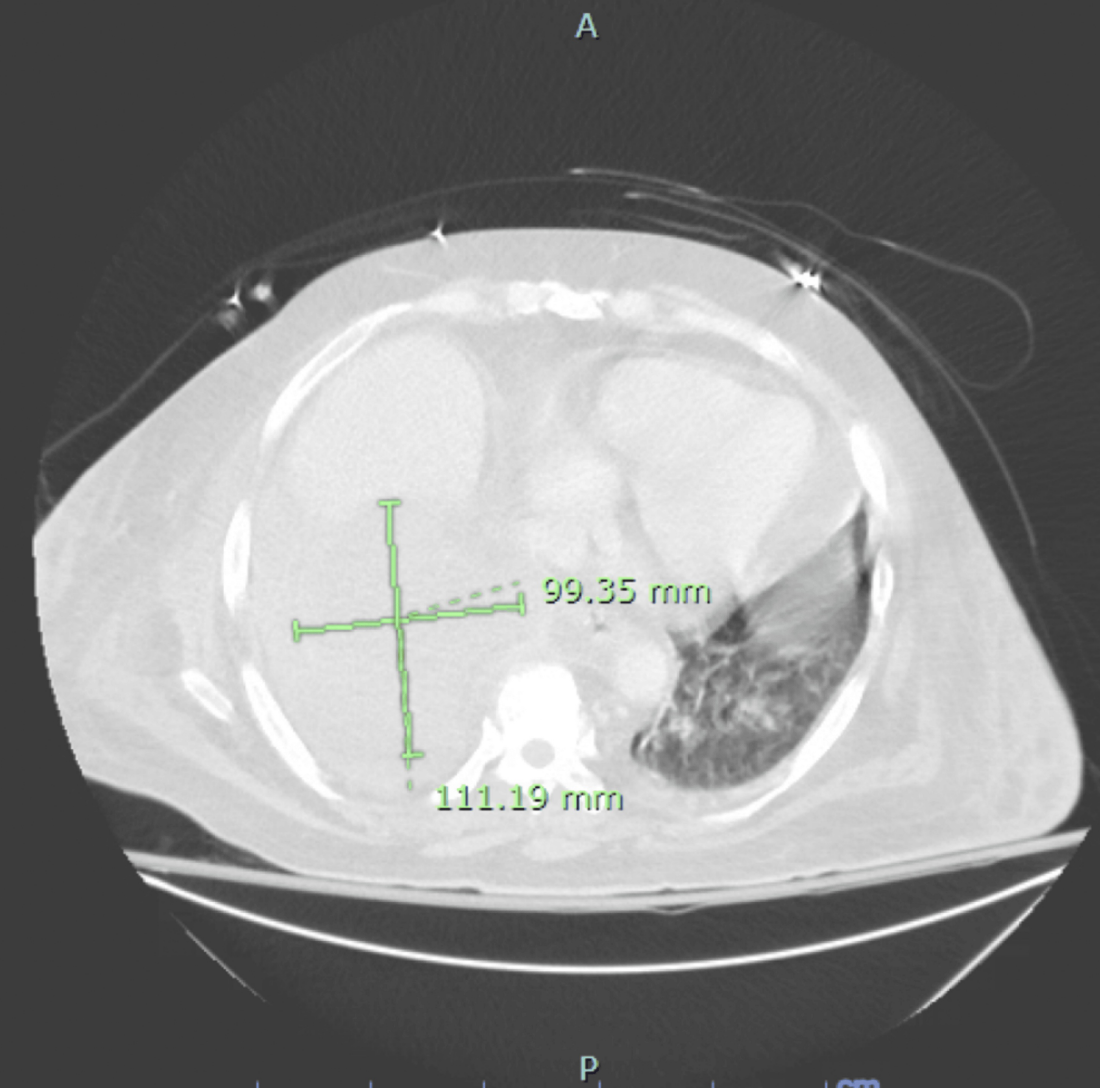
Progression of the cavitary lesion on day 12, now showing collapse of the right lower and middle lobes, invasion of the diaphragm, and mass effect on the liver. Image obtained at our institution, Baystate Medical Center, and used with family consent.

On hospital day 9, the patient began experiencing episodes of respiratory distress, which waxed and waned over the subsequent five days and required intermittent noninvasive ventilation. He remained febrile with a maximum recorded temperature of 100.7°F. A repeat CT on hospital day 12 showed worsening of the right lung consolidation now involving the right lower, middle, and upper lobes. A new pleural effusion obscured the cavitary lesion in the lung. On hospital day 13, the patient underwent pleural fluid drainage of approximately 900 cc of exudative fluid and chest tube placement. No pleural fluid culture was sent. On day 14, a CT with intravenous contrast showed a large non-enhancing structure in the RLL with complete collapse of the right lower and middle lobes. There was additional invasion into the diaphragm and abdomen with a mass effect on the liver (Figure 2).

**Figure 3:**
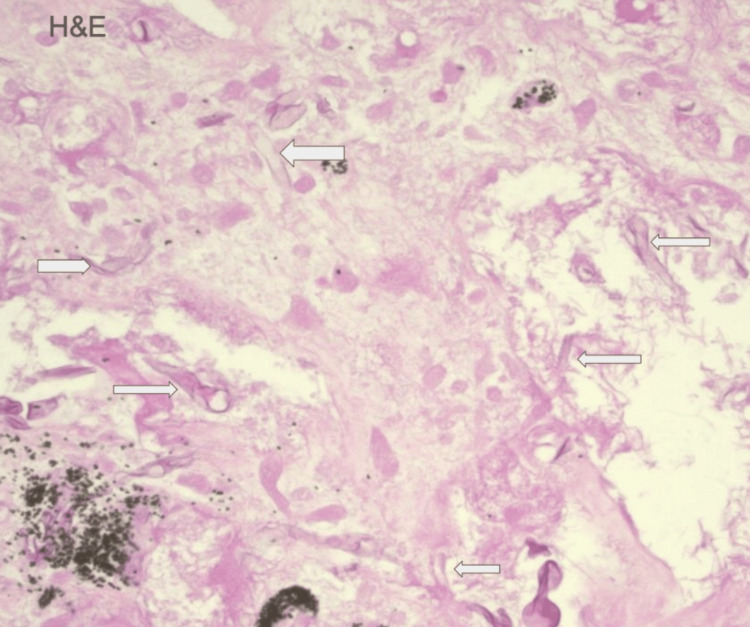
Histology of the right core mass lung tissue stained with hematoxylin and eosin, revealing right-angled, broad, aseptate, ribbon-like hyphae consistent with Mucor (arrows). Tissue analyzed by the Department of Pathology at Baystate Medical Center at 100x. Image obtained at our institution, Baystate Medical Center, and used with family consent.

On day 18, a CT-guided biopsy of the RLL mass was performed. Histology revealed fungal elements with morphology consistent with mucor (Figure 3). Liposomal amphotericin B was initiated. The patient was transferred to an outside facility but died before surgical debridement could be performed.

## Discussion

Renal transplant recipients are at increased risk of both opportunistic infections and malignancies due to immunosuppression. Mucormycosis is a rare but life-threatening infection accounting for up to 2% of all fungal infections in renal transplant recipients [3]. Mucor presents in three clinical patterns: rhino-cerebral mucormycosis, PM, and disseminated mucormycosis. PM is less common and associated with higher-risk clinical settings, including the presence of hematological malignancies, diabetes mellitus, and solid organ or hematopoietic stem cell transplantation [4,5].

Systemic or disseminated mucormycosis is rare but can involve the gastrointestinal tract and the kidneys, with the highest mortality rate [6]. In this case, the patient presented with PM. Mucormycosis grows on decaying food, animal excrement, and soil and is transmitted through inhalation of spores. Demographically, mucor infections are more common in males, accounting for 70.97% of reported cases [7].

Invasive fungal infections tend to occur during periods of higher levels of immunosuppression, typically early post-transplant, with a median time from renal transplantation to diagnosis of six months [8]. Symptoms are nonspecific and include fevers, dyspnea, pleuritic pain, and nonproductive cough; hemoptysis is common and suggests vascular invasion, which can be fatal [9]. PM presenting with concomitant bacterial pneumonia with *Streptococcus* or *Staphylococcus* organisms, as in this case, occurs in 30% of cases, often delaying diagnosis [3].

Another common pitfall leading to delayed diagnosis is the mistaken assumption that negative galactomannan and beta-D-glucan tests rule out fungal infection. Although both are polysaccharides, they differ in structure and location. β-D-glucan consists of linear glucose polymers linked by glycosidic bonds and is a component of the cell walls of many fungi and yeasts, whereas galactomannan, composed of a galactofuranose-mannose backbone, is primarily found in the cell walls of *Aspergillus* species [10,11]. The diagnostic performance of β-D-glucan is highly dependent on pretest probability. In a multicenter study evaluating the Fungitell assay (cutoff 80 pg/mL), the negative predictive value was 73%, and the positive predictive value was 89% [12]. It is to note that beta-D-glucan may be negative in infections caused by organisms such as *Mucor*, *Rhizopus*, and *Cryptococcus*, which lack significant amounts of this cell wall component. Therefore, particularly in patients with high clinical suspicion, a negative β-D-glucan result should not be used to exclude fungal infection or delay pursuit of definitive tissue diagnosis.

**Figure 4:**
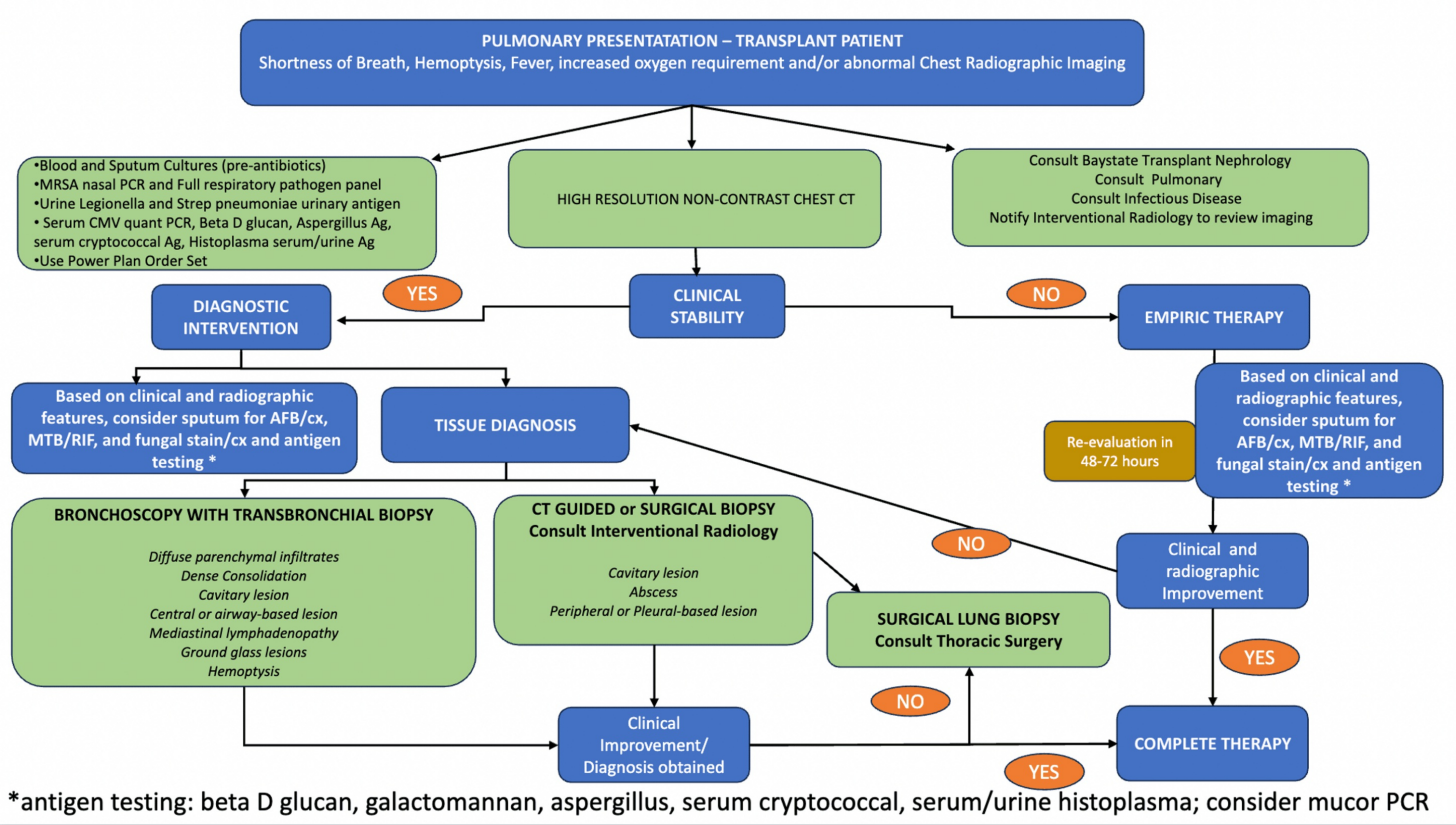
Algorithm for pulmonary presentation in a renal transplant recipient. This figure illustrates the institutional workflow for evaluating transplant patients presenting with pulmonary symptoms, including initial imaging, assessment of clinical stability, targeted microbiologic testing, and escalation from bronchoscopy to CT-guided or surgical lung biopsy when indicated. CT: computed tomography; MRSA: methicillin-resistant *Staphylococcus aureus*; PCR: polymerase chain reaction; CMV: cytomegalovirus; Strep: *Streptococcus*; Ag: *Aspergillus*; AFB: acid-fast bacilli; MTB: *Mycobacterium tuberculosis*; RIF: rifampicin Source: Figure created by the authors using Microsoft Word (Microsoft Corp., Redmond, WA, USA).

Similarly, galactomannan is an antigen test for *Aspergillus* species and does not detect mucormycosis [13]. To prevent delays in diagnosis, it is necessary to maintain a heightened level of suspicion for fungal organisms when the clinical course is not straightforward. For patients who fail to respond to empiric therapy for bacterial pneumonia, broadening the differential diagnosis and reassessing for other opportunistic infections is critically important, including reculturing body fluids, reimaging, and more aggressive diagnostic approaches. A lung biopsy is recommended if the patient remains unresponsive to treatment because definitive fungal identification requires either a positive culture or a histologic diagnosis [14,15]. Biopsy samples stained with hematoxylin and eosin in cases of mucor infection will show non-septate, right-angled branching hyphae, which differentiates it from the septated, dichotomous branching pattern of *Aspergillus* species [14,15]. When cultures are delayed or negative, identifying mucormycosis DNA by PCR in tissue, blood, or fluid samples effectively confirms the diagnosis [16]. However, obtaining PCR results can take up to a week if not performed by a local laboratory. A structured diagnostic approach for pulmonary presentations in renal transplant recipients is summarized in Figure 4.

Furthermore, there are some radiological features that should increase suspicion for PM. Although most features are nonspecific and can range from solitary nodules and ground-glass opacities early in the course to multilobar consolidation and multiple nodules or masses, the presence of a reverse halo sign on a lung CT scan should raise suspicion for invasive fungal infection such as mucormycosis [16,17]. Characterized by a central area of ground-glass opacity encircled by consolidation, the reverse halo sign, if present, predicts a poor prognosis if surgical therapy is delayed [10]. Lastly, rapid radiologic progression, including three-lobe pulmonary involvement, should prompt rapid tissue sampling for invasive fungal infections.

Treatment of PM requires administering intravenous liposomal amphotericin B. Other azole antifungal agents, like voriconazole, exhibit limited activity against mucormycosis [18]. Posaconazole, a newer broad-spectrum triazole, while not FDA-approved, serves as a second-line treatment with a success rate ranging between 65% and 70% [18]. Isavucanazonium sulfate, a prodrug of isavuconazole, is FDA-approved; however, there is limited comparative data assessing its efficacy compared to amphotericin [18]. Recent studies suggest that surgical debridement complements the antifungal treatment regimen, as the extensive lung necrosis associated with invasive mucor can limit antifungal penetration. Another rationale for adding surgical resection to medical intervention is the tendency of mucor to infiltrate pulmonary vasculature, often leading to life-threatening hemoptysis. Excising the infected tissue surgically is crucial for successful treatment [17,19,20]. A meta-analysis including 18 studies demonstrated significantly better mortality rates for combined medical-surgical treatment versus medical therapy alone [21].

## Conclusions

This case highlights the critical need for swift and targeted diagnostic measures when managing pulmonary infections in immunocompromised individuals. If empiric bacterial antibiotic therapy proves ineffective within the initial days and serologies are unrevealing, prompt histological diagnosis is needed to target therapy for potentially fatal fungal infections like PM. Clinical deterioration and rapid disease progression on lung CT scans in such patients should trigger suspicion for this rare infection. Additionally, it is essential to recognize that a negative beta-D-glucan result does not rule out mucormycosis infection; instead, it should heighten suspicion when imaging and clinical course suggest invasive opportunistic infection. Given the vulnerability of immunocompromised patients to rarer opportunistic infections, a proactive and aggressive diagnostic stance is essential to optimize outcomes.
